# Research and Remedies

**Published:** 1912-11-30

**Authors:** 


### RESEARCH AND REMEDIES.

### Gastric and Duodenal Ulcer.

A careful study of 1,000 cases of gastric and duodenal ulcer has been published in the *American Journal of the Medical Sciences* by Friedenwald. His conclusions are much the same as those of the leading British authorities upon this subject; but it is worth while to repeat some of them, since they present so very different a picture to that which was currently believed by the medical profession a few years ago. More than twice as many males as females suffer from these ulcers. In almost half the total number a history of over-indulgence in food or drink can be elicited. Anæmia is present in a large proportion of the cases. Hyperacidity of the stomach contents is proportionately commoner in males than in females, and in acute than in chronic cases; but, as a matter of fact, it is only found in 30 per cent. of cases. Subacidity is present in 23 per cent., and normal acidity in 46 per cent. Pain occurs in 94 per cent., and is most frequent in association with high acidity. Long periods of intermission of this symptom, as well as of the other symptoms, may occur. An epigastric area of tenderness is found in 90 per cent.; a dorsal area in 32 per cent. Vomiting occurs in 67 per cent. Hæmatemesis is a symptom of 22 per cent. of cases, and melæna of 51 per cent. Occult melæna is found in 81 per cent. Duodenal ulcer is more common than the gastric kind, especially so in males; pain occurs in 96.5 per cent. of duodenal ulcers, in which also long periods of intermission of all symptoms are especially common.

